# General Practitioners are poor at identifying the eating disorders

**DOI:** 10.1080/21662630.2013.859437

**Published:** 2014-01-06

**Authors:** Glenn Waller, Nadia Micali, Alison James

**Affiliations:** a Department of Psychology, University of Sheffield, Sheffield, UK; b Behavioural and Brain Sciences Unit, Institute of Child health, University College London, London, UK; c University Health Service, University of Sheffield, Sheffield, UK

## General Practitioners are poor at identifying and treating the eating disorders: Glenn

The very first thing I should say is that I think that General Practitioners (GPs, or family physicians, in case the term baffles) are a fine bunch of people, as a whole. This is the same thing that I would say about most groups of healthcare workers (including my own profession). The work that GPs do is demanding and takes a much broader knowledge and skills base than most professionals will ever need or achieve. They are the face of the healthcare system for most patients and the pressure on them is immense. Indeed, in the UK, they are now being tasked with commissioning healthcare too – as if the demands of delivering and channelling physical and mental healthcare for a nation were not sufficient …

Now, usually in a debate, praise of this sort for GPs should be followed by condemnation of the entire profession. That is not going to happen here. I really do not know how GPs manage the diversity that comes their way as well as they do. However, there are very clear issues that are inherent in their generalist role – just how good can one be at identifying or responding to unusual disorders that the patient is often loath to bring along? I am not claiming that that there is anything special about the eating disorders (EDs) – I suspect that the same would apply to the identification and treatment of any relatively rare disorder. When one works with the EDs, it is easy to forget that they are rather uncommon relative to other physical and mental health problems.

My intention here is not to argue that GPs should be more aware of the EDs and what to do about them, but to consider why GPs are not more aware and how real life and entirely human biases affect their actions. I intend to bring in research evidence, clinical experience and a touch of the obvious to convince you that whatever Nadia says to try to counter this proposal, her case will be extraordinarily optimistic.

### The evidence

First, do all GPs identify cases equally? No. In a survey of over a 1000 GPs in the UK, Currin (2006) has shown that half report seeing no new cases per year, while others see a mean of two. This discrepancy has to be seen in the context of the fact that both numbers are probably well below what one would expect from population incidence figures. Furthermore, simply having the symptoms of an ED is not enough. In a vignette study completed by 154 GPs, Currin et al. (2007) have shown they gave a diagnosis of an ED to under 70% of cases with clear ED presentation. Even low body mass index (BMI) did not seem to trigger a clear clinical decision for about half of GPs – and the resulting ‘wait and see’ approach did not yield much clarity. Similarly, Lask et al. (2005) showed that it can take GPs a while to spot the meaning of a young patient attending to consult about eating and weight concerns. If we were astrophysicists, we would be worrying about all the dark matter – we know that the cases must be out there somewhere, but we are having trouble seeing them.

So, if you have an ED and you want to be identified by your GP or passed on for treatment, what does the evidence say that you should you do? To enhance your chances of being spotted, try to make sure that you are female, particularly if you have Bulimia Nervosa (BN) (men with the same symptoms are far more likely to be diagnosed as depressed – Currin et al., 2007). Having been spotted, if you want to be referred on for treatment, then try to live relatively near to a specialist centre, register at a practice with more GPs, and have a doctor who has specific GP training and accreditation (Hugo, Kendrick, Reid, & Lacey, 2000). The gender of your GP is linked to referral patterns, but in different ways for different disorders in different studies (Feeney, Noble, & Waller, 2007; Hugo et al., 2000).

Let us assume that you *are* spotted by your GP – how prepared is he or she to plan your care from that point? Again, think about the numbers of guidelines that GPs receive for a variety of disorders – they are forced to prioritise, and it makes sense to do so in a way that maximises patient care. Nearly all GPs are aware of National Institute for Clinical Excellence (NICE) guidelines for depression and three-quarters have read them (Gyani, Pumphrey, Parker, Shafran, & Rose, 2012a). Nearly half are aware of the NICE guidelines for obsessive-compulsive disorder and 30% have read them (Gyani, Shafran, & Rose, 2012b). However, under 4% of GPs report using any guidelines at all for the EDs (Currin et al., 2007). While the Currin paper shows that GPs do some of the things in the guidelines, they do them on the basis of clinical judgement (and in a patchy fashion) rather than following guidelines. No wonder that some recommendations (e.g. referral to specialist services at particular BMIs) are not followed by over half of GPs when deciding how to treat the EDs.

### Experience

However, while the evidence is overwhelmingly in favour of the motion, I do not want to be unfair. As Nadia will not have much research to call on in support of her case, it is important that we consider our experience of GPs who we have worked with. I have worked with great GPs, who really see the importance of identifying and responding to the EDs, but I have also had contact with GPs who appear to be resistant to the idea that the patient might have an ED or any physical risk and who certainly do not want any responsibility for the patient (whatever NICE guidelines might say about their role).

This is not just about GPs who I have worked with. At assessment or starting treatment, I always ask about the route the patient took to get in order to see a specialist and tales unfold of some fantastic GPs, some pressured GPs, some baffled GPs, and a few GPs who appear to be hostile. In short, we find the patchiness that one would expect when asking busy professionals to do things under pressure. So my experiences are, in brief:

- many patients report that the GP misses hints over the course of multiple visits (and, being fair, the patients admit that the hints are pretty obscure in lots of cases);
- many GPs do not know what the best options are (though a fair number do) and do not always know about specialist services. Many patients report asking for a referral on multiple occasions but the GP being unaware that there was a service to refer to;
- some GPs offer ineffective or potentially damaging treatments (e.g. treating bone density issues with the contraceptive pill or even bisphosphonates);
- a lot of GPs play the ‘watching brief ‘ role (which can be a sensible thing to do), but then panic and push for immediate in-patient admission (even when it would be pointless);
- a few GPs are easily persuaded to carry out test after test in order to check what is physically wrong with the patient and appear to be more inclined to believe the patient's account of eating normally or wanting to eat normally, even when the patient's family are pushing the notion of an ED; and
- a (mercifully) few GPs appear to dislike eating-disordered patients or to trivialise the problems (but that does not distinguish GPs – so do a lot of other clinicians).

### The obvious

The average GP consultation is a few minutes long, with little or no preparation time. Many patients with EDs are ashamed, do not think that they have a problem or do not want anyone interfering. Therefore, they report other problems, possibly hoping that the GP will spot the real issue. So the GP has a few minutes to bond with the patient, deal with the reported problem, identify the underlying eating problem, review the case history, consider treatment options, plan a course of action, write a prescription or make an onward referral, explain the process to the patient, apologise for delays, motivate the patient to do the necessary work, and get the patient out of the door. Breathing appears to be secondary. So when is there time to consider what are, for the GP, relatively rare options such as EDs? The GP could reasonably argue that such things are the role of specialists.

So why not use more time-efficient methods? Certainly, there are screening measures, designed for use in primary care, but they tend to be insufficiently specific. Using them, if 1 patient in 10 shows up as having a potential ED (and that is a low rate, given the properties of the measures), but only 1 in 10 of those actually has a problem, the GP now has the joy of writing 10 referral letters for every 100 patients seen, in order to get 1 true patient seen by a specialist service (if that patient ever does get seen, given all the non-cases that the specialist services are now drowning in as a result of this approach). Similarly, there are guided self-help methods that the GP can successfully employ with patients. But does the GP really have time to do this properly or is it more likely to end up as the (far) less effective unguided self-help?

When the problem becomes clear or was obvious all along (especially when the patient is very thin), the GP often has additional problems in getting the patient to treatment (e.g. the patient arguing that her or his difficulty is biological and the family being conflicted over the desire for urgent treatment while not wanting it to get in the way of their child's studies). But greater than all of these is the problem of what to with the patient. Is there a local tertiary EDs service? Is the GP allowed to refer directly? Do secondary services actually see patients with EDs (for example, the ‘Improving Access to Psychological Therapies’ services in the UK explicitly exclude the EDs)? Just how long will the waiting lists be? And finally, when the patient gets to the appropriate service and what are the chances that an effective therapy will be offered? Along with carers, the GP is going to be carrying a lot of the burden of care and support throughout this process. That is a lot of short consultations. A lot.

### Summary (so far)

I would love to say that GPs are perfect, and that they identify and treat all rare cases rapidly and effectively, but I would also like to be better at hanging pictures straight. There are some things that are just not going to happen. Why should GPs not be better at identifying and treating the EDs? The clue is in the name – *General* Practitioner. Healthcare depends on there being frontline clinicians who are generalists rather than specialists. Even where a GP has a special interest, why should that be in the EDs? There are a lot of other special topics.

Consequently, I would like you to consider the three elements that I have outlined here – the research, the evidence of patients’ (and your own) experience, and the obvious. Then accept that it is inevitable that you will agree with the motion – GPs are poor at identifying and treating the EDs.

So what can we do about this? Well, I suspect that I will be coming back to that after Nadia has had her say. The obvious things to suggest are issues such as training or devolving this task to other team members, so watch this space post-Nadia.

## GPs are not poor at identifying the EDs: Nadia

The nature of debating implies a critical rebuttal of Glenn's views and I assume I should fiercely disagree with the proposer. However, given that scarcity of evidence in the field my job is rather arduous. Therefore, I would like to respond to the motion with three main points:

(1) Why GPs *might not be so* poor at identifying the EDs.
(2) They might actually have become better at recognising ED.
(3) And if identification is poor, why are we not doing anything about it.

### Why GPs might not be so poor at identifying the EDs

The GPs in the UK have an important role as gatekeepers of access to secondary and tertiary care. Given that they are generalists, the GP training curriculums also includes mental health (http://www.rcgp.org.uk). Indeed, several GPs train in psychiatry for six months during their training.

During my career as a consultant psychiatrist in EDs, I have met many GPs who were very involved in the management of their ED patient and even attended planning meetings for their ED patients admitted to an in-patient unit.

Studies across Europe, the USA, and Australia have highlighted that patients with ED are more likely to attend primary care prior to a diagnosis and following an ED diagnosis (Mond, Myers, Crosby, Hay, & Mitchell, 2010; Ogg, Millar, Pusztai, & Thom, 1997; Van Son, Hoek, Van Hoeken, Schellevis, & Van Furth, 2012); although patients with BN were more likely to see their GP for problems other than their eating.

Unfortunately, research on GPs knowledge and attitudes to ED has been and is scant. Currin et al. (2007) did show that amongst the 82 GPs they obtained data from there were gaps in knowledge of ED. However, interestingly GP's knowledge and attitudes did not predict how likely they were to diagnose an ED.

In a small qualitative study, Reid, Williams, and Hammersley (2010) identified concerns by the GPs interviewed about being able to recognise and manage ED, although they reported more difficulties in managing rather than diagnosing ED. A similar small qualitative study of six GPs similarly identified a lack of confidence in GPs interviewed when distinguishing Anorexia Nervosa (AN) from less pathological disordered eating and using a diagnosis of ED rather than AN to ‘label’ patients in some cases (Hunt & Churchill, 2013).

Most studies seem in fact to suggest that GPs (or family doctors) are more comfortable in diagnosing an ED if a specialist service is available in their area or if they serve a large student population (and hence are exposed to a larger number of ED patients) (Banas, Redfern, Wanjiku, Lazebnik, & Rome, 2013; Reid et al., 2010). This might suggest a pattern of under-diagnosis in some geographical areas rather than under-recognition and better recognition and identification in other areas.

However, diagnoses by GP are relatively reliable. In a study by Turnbull, Ward, Treasure, Jick, and Derby (1996), using data from the general practice research database (GPRD) the positive predictive value for a diagnosis of AN or BN was above 90%, which is extremely good. Amongst cases referred to an outpatient ED service in Australia, GPs – who were the majority of referrers (82%) 65% of cases were correctly diagnosed by GPs (as compared to the eating disorders examination [EDE]), with the main difference being an under-recognition of Eating Disorder Not Otherwise Specified (EDNOS) by GPs, but overall GPs were able to identify AN-like and BN-like disorders (Allen, Fursland, Watson, & Byrne, 2011). Given that as specialists we also struggle with the notion of what is EDNOS, I believe that is pretty good going.

This leads me to a related important point, i.e. What do we want from GPs? What would the preferred outcome be of a consultation between a GP and a patient with an ED? Would we want the GP to diagnose an ED? Or to suspect an ED and take all the necessary steps towards confirming the diagnosis? My vote would be for the latter. Excellence in diagnosing ED might not be achievable or desirable, but awareness and knowledge of how an ED manifests is important.

The EDs have a complex manifestation, including denial of symptoms and difficulties in acknowledging problematic behaviours, and a high rate of comorbidities. Across the qualitative studies cited above GPs described finding it frustrating to deal with these additional hurdles.

Differences in recognition of ED patients might also exist across ages. In fact, it is likely that adolescent EDs might be ‘easier’ to diagnose compared to adults given parental concerns and parental report of symptoms. I am unaware of any study that has investigated detection rates in primary care of adolescents vs. adults with ED.

I would therefore argue that GPs are able to suspect an ED and diagnose it, even though they might be more au fait with classical ED nomenclature (AN and BN) rather than EDNOS.

As Glenn points out, however, initial management of the ED might be a different story, and although NICE guidelines recommend that GPs plan treatment including onward referral, this might not always be the case. In a small qualitative study focusing on AN, GPs highlighted two sides to this process, despite the GP being confident about the patient needing a referral, the difficulties of the patients themselves accepting a referral to specialist care and attending their appointments in specialist care (Hunt & Churchill, 2013). A recent study on care pathways highlighted differences in number of cases of adolescent EDs presenting to secondary/tertiary care per year in areas with specialist ED services or specialist Child and Adolescent Mental Health Services (CAMHS) compared to non-specialist CAMHS, suggesting that GPs might be less likely to refer onwards if they do not have a specialist team available in their area (House et al., 2012).

Several of the above studies have shown that participants (GPs) reported a desire for training in EDs (Banas et al., 2013; Hunt & Churchill, 2013; Linville, Brown, & O'Neil, 2012; Reid et al., 2010). Several courses for GPs are available in the UK and Australia.

This brings me onto my next point, although a couple of studies cited above are recent, knowledge and awareness of EDs among GP is likely to be continuously increasing.

### They might actually have become better at recognising ED

Our recent study investigating the incidence of EDs in primary care in the UK identified an increase in the number of males and females being diagnosed with an ED in primary care between 2000 and 2009. This increase was mainly due to a significant increase of EDNOS diagnoses (Micali, Hagberg, Petersen, & Treasure, 2013). This increase could well be explained by increased knowledge/awareness of atypical EDs. Similarly, Turnbull's study on the incidence of ED in the GPRD in the early 1990s (1996) showed an increase in the incidence of BN that stabilised in the late 1990s. Although our study used electronic records from the GPRD, where GPs incorporate data from secondary or tertiary care when patients are referred both studies suggest that GPs might have improved in diagnosing ED over time.

### Can we do more to improve identification?

Improved identification, we all hope, will lead to improved outcomes. If treated early ED, especially in adolescence can result in better outcomes, therefore early recognition is important.

However, some authors have underlined that improving recognition of all mental health disorders in primary care might not be possible and that in fact identification rates of mental health disorders are not very different from those of physical disorders (Wittchen, Mühlig, & Beesdo, 2003). They also argue that failed recognition might not only be due to GP's poor knowledge, but to a natural threshold in illness severity, whereby GPs will identify disorders that are above that threshold. Another important issue is the direct lack of evidence that improved recognition leads to better outcomes.

What could actually lead to better recognition is debatable. The NICE guidelines, for example, suggest using the SCOFF (Morgan, Reid, & Lacey, 1999) as a screening tool for ED in primary care. However, reported specificity, sensitivity, and positive predictive value of the SCOFF in primary care vary across studies (Hill, Reid, Morgan, & Leacy, 2010; Mond et al., 2008). Moreover, its psychometric properties across genders need further study.

To summarise: I have highlighted supported evidence that GPs are not bad at diagnosing ED, although they have concerns about their skills in identifying ED. Moreover, studies from large GP databases show that in fact GPs might continuously be getting better at identifying ED. Although knowledge is important, the studies reviewed above all suggest that a diagnosis of ED in primary care depends not only on knowledge. It would be irresponsible not to consider all the other factors that might lead a GP to diagnose an ED, including availability of services to refer to, individual patient characteristic, and patient's age, amongst others. Finally, I would like to reiterate two main points: (1) diagnosis as such might not be what is required by a GP, but some understanding that the patient they are faced with probably has an ED that is severe enough to warrant further investigation/treatment might be the ideal skill and (2) identification as such is meaningless without a cascade of treatment options/services following on from a diagnosis.

## A bit of a rebuttal: Glenn

Do not you just hate it when a debate sort of melts into general agreement? No fist fights between the participants (as was alleged to have happened in the most recent London mayoral campaign). Just everyone sort of agreeing about the key points.

Now, you might be thinking that you missed a bit out – agreement? But I want to consider (briefly) why I think that Nadia and I agree far more than we disagree. Then I will consider what we might be able to do about the identification issue within the real world, before handing you back to Nadia and then Alison – two voices of reason are always better than one. So, considering Nadia's points in order, as I see them.

First, I agree totally with Nadia – GPs *might* be better at identifying cases than I allowed. However, the research and experience that she cites do not really address the issue of identification in any way that makes me more optimistic. For example, the findings of Hunt and Churchill (2013) give an idea of how complex the issue of diagnosis is, both clinically and interpersonally – answer: ‘very'. As I said earlier, some GPs are great at this work and I have met a good number of that sort. I agree with Nadia again – there are reasons why some GPs are better (local specialist service and lots of experience). However, there are many more who never make contact beyond making a referral letter (if they even do that). In addition, the GPs who we meet in highly specialist services are not necessarily that common or representative. And what does it say when Nadia notes that some GPs even attend planning meetings? It says ‘exception rather than rule'. I repeat myself – GPs are extremely busy people and they have lots of potential special interests. We cannot count on their being focused on the EDs. So while they *might* be getting better at spotting cases, there is evidence that they *are*.

The next point where I agree with Nadia is that GP diagnoses of the EDs are fairly reliable, though not perfectly (for example, I also agree that to expect GPs to be good at identifying EDNOS would be unrealistic). However, reliability is not validity, and it is validity that counts here. In the GP research database, the diagnosis is checked against the GP's identification of it. What if the GP did not identify it in the first place? For example, let us assume that my brother and I have the same relatively rare disorder. My brother tells his GP, but I do not (though I might try dropping a few hints, only to be frustrated when they are not picked up). Then a researcher comes along and examines GP records of known cases to see if the diagnosis of this disorder is accurate in my brother's case, and it usually is. In calculating prevalence that is an index of *reliability* of case identification. However, that does not mean that the count of this disorder in the GP's records is an accurate reflection of that disorder's prevalence, because my own case is not noted anywhere. That would be the index of *validity* of prevalence. Incidentally, calculating positive predictive values is a complex job, with assumptions that are not always clear. OK – technical stuff over for the moment.

I agree that diagnosis of an ED should start with a hunch on the part of the GP, even if the confirmation happens elsewhere. I certainly hope that it is easier to spot adolescent cases, as Nadia suggests. Where I do not fully buy Nadia's argument is when she says that GPs are able to suspect and diagnose an ED, but leaves hanging the implication that they will, therefore, do so. Sufferers need to come to the GP, the patient has to tell the GP about symptoms that might be masked by shame and personal control, and the disorder has to be separated from other possibilities. Remember that only about one sufferer in six or seven has AN, many of those patients dress to mask their thinness and not all thin people have AN. Suddenly, the GP's job just got a lot harder and the hunch remains untriggered.

My next point of agreement with Nadia is that GPs *might* have become better at recognising the EDs. That ‘might’ word again … However, I am being unfair there, because I am pretty certain that GP's have got better at identifying cases. Whether that is a product of there being more cases in the world or better identification of existing cases is a moot point, as different authors seem to take the same findings to indicate either a rising tide of people developing EDs or better GP identification. The papers that Nadia describes talk about incidence, but are they really talking about that? If there really is an increase in incidence, then to identify more cases might indicate that our skills of identification are no better. Purely hypothetically, if there were 1000 cases in an area in 1995 and 1200 in 2005, then to identify 200 cases in 1995 and 240 cases in 2005 simply means that the identification rate has stayed stable at 20%. So my agreement with Nadia is based on gut instinct – GPs might indeed be getting better at identifying cases. However, the same findings are explained differently in the literature. So I am going out on a limb to agree with Nadia here, but it is a limb that I am happy to perch on with her, based on my best guess. What I do not know is how far we have to go before GPs can be seen as identifying a good proportion of cases on their registers.

Finally, what can we do to improve GPs’ ability to identify and treat cases? Nadia and I are as one about the importance of early identification. It seems so natural to want to identify case speedily, in order to get treatment going sooner (though there is only preliminary and partial evidence about the effectiveness of this approach to date). However, maybe we could go a step or two beyond Nadia's apparent pessimism on the subject of improving GP performance in this domain. As Nadia points out, enhancing knowledge might not be enough, especially when the disorder is relatively rare. Furthermore, it is very difficult to get hard-pressed GPs to attend top-up training. Therefore, I would suggest that GPs’ original training should be standardised to include a simple teaching module, including:

- the diversity of cases (age, gender, ethnicity, etc.);
- the fact that most cases are not underweight;
- simple ‘hunch-level’ questions about case identification (rather than the more complex issue of specifying diagnosis and certainly not an under-specific questionnaire);
- a list of the urgent symptoms to look out for (e.g. muscular weakness); and
- references to self-help material, with more on how and when to seek specialist help.

That could be done on one side of a piece of paper, rather than adding to the number of comprehensive guidelines that GPs do not have the time to read.

Next, how about having a poster in every GP clinic (and dental practice – dentists are often the first professionals to spot patients with a bulimic disorder) suggesting that patients or carers should raise their own concerns with the GPs or with a specified practice nurse? That poster should be clear about the EDs being diverse, so that patients do not fall into the same trap as professionals – assuming that you only have an ED if you are underweight. Consultation with self-help organisations would be likely to inform how this poster could be most effectively presented and distributed.

Finally, let us learn from existing practice when it comes to getting health promotion to happen. Part of GPs' funding comes from making sure that a proportion of patients receive specific services – health checks for new patients, inoculations, screening, etc. How about adding screening for the EDs to that process, so that the GP or a practice nurse uses the ‘ hunch’ questions? The business model is one that has worked elsewhere. However, Nadia is right once more – GPs will have little interest in simply identifying cases and offering limited help. There has to be an infrastructure of services offering further treatment options, which are easy to access and which keep the GP informed about the patient's progress.

## How do we move forward: Nadia

Glenn has very clearly highlighted how despite starting from opposing views our premises overlap, hence I will change tack and focus my response on how to address what we both understand as limitations of current GP knowledge and identification of ED and potential obstacles.

We have so far established that GPs vary in their ability to identify and recognise ED; that some characteristics (individual interest, experience and training, and practice location) might influence their ability to identify ED; and that there might have been some improvements over the last decade. We have also established that identification might be a pointless exercise if it is not integrated within set care pathways and availability of relevant services.

Our field is certainly not the first to grapple with issues of a chronic disease, affecting mental and physical health of an individual, that is common but poorly recognised. It seems to me the depression field went through this process 10 or more years ago. Since then evidence on the prevalence of depression, scientific advances, and the widespread availability of good treatment (at various service tiers), i.e. including the drive to increase access to psychological therapies introduced with ‘Improved Access to Psychological Services’ in the UK has led to increased public awareness, introduction of screening programmes (i.e. the Whooley questions introduced and recommended by NICE guidelines as a screener for post-natal depression) (Agency for Healthcare Research and Quality, 2003; Pignone et al., 2002), and improved understanding and recognition of depression. We are behind as a field and this is a threat and an opportunity.

Glenn focuses on three main areas where changes might be possible in relation to ED: training, self-help materials, and screening.

Both Glenn and I have commented on difficulties inherent in adding to someone's professional training when there is already an overload of guidelines, recommendations, and yet-more training courses. However, as Glenn points out training in primary care could be directed not just at busy GPs but also practice nurses.

Moreover, focused training on broad ED characteristics that are likely to be relevant to more patients, such as which groups are at high risk, symptoms and physical signs that are common to all ED might be more achievable and desirable than an in-depth knowledge of ED. As Glenn suggests, this broad training could be supplemented by more detailed material (online, posters, etc.).

Self-help materials have become increasingly popular and used in mental health. A poster or leaflet available to all patients in GP waiting rooms or antenatal clinics or dental practices could be a useful tool to de-stigmatise EDs and allow sufferers to ask for help. Similarly, a leaflet for parents about EDs symptoms in adolescents and how to ask for help might be a very useful tool to improve identification in primary care.

Screening is a delicate subject, with rather polarised views amongst ED practitioners about what should or should not be screened for. Considerations about the impact of false positives and false negatives at the individual and service level and its ethical implications need to be taken into account. However, I believe that for EDs the benefits of screening (high-risk subjects possibly) would outweigh most downsides. Criteria for screening were set out in the 1960 s by Wilson and Jungner (1968) and are still used. These are:

(1) The condition sought should be an important health problem.
(2) There should be an accepted treatment for patients with recognised disease.
(3) Facilities for diagnosis and treatment should be available.
(4) There should be a recognisable latent or early symptomatic stage.
(5) There should be a suitable test or examination.
(6) The test should be acceptable to the population.
(7) The natural history of the condition, including development from latent to declared disease, should be adequately understood.
(8) There should be an agreed policy on whom to treat as patients.
(9) The cost of case-finding (including diagnosis and treatment of patients diagnosed) should be economically balanced in relation to possible expenditure on medical care as a whole.
(10) Case-finding should be a continuing process and not a ‘once and for all’ project.

We could spend days debating whether EDs meet each of these criteria; however, you will surely all agree EDs broadly meet most (if not all) criteria.

Furthermore, new advances in technology make screening even more feasible (thanks to tablets and touchscreen stations at GP surgeries amongst others) and have been used to screen for lifestyle factors (smoking, alcohol and drug use, and mental health problems) (Goodyear-Smith, Warren, Bojic, & Chong, 2013; Paul et al., 2013). So why not EDs?

Two aspects need further consideration: one is about feasibility and acceptability of screening and second, and I hate to say the same thing again, is what happens following screening. Do we, at this stage, have enough evidence regarding the feasibility, acceptability, cost, benefit, and cost/benefit ratio to patients and health care professional of available identification methods for ED? One of the few studies available to have focused on acceptability and feasibility of screening for ED, that used the SCOFF as a screening instrument showed that screening for ED was seen as acceptable and feasible by healthcare professionals, and about half of the women approached did complete the screening (Johnston, Fornai, Cabrini, & Kendrick, 2007). However, moving onto my second concern, not all GPs took action following screening for patients who showed positive, although screening was seen as acceptable (Johnston et al., 2007). This takes us back to the issue highlighted by Glenn and I and shown in qualitative studies that without adequate referral pathways and accessible services there is almost no point in implementing a screening programme.

Overall, it seems to me that the issue of identification of ED is truly a jigsaw puzzle and several pieces need to come together to effectively strengthen and improve recognition of ED in primary care and that although some pieces might be already set in the right place, several more need to be ‘set in place’.

## GPs are poor at identifying and treating EDs. Commentary: Alison James

You might at this point be expecting a spirited defence of General Practice and an assertion that we GPs are competent and confident at identifying and treating EDs. Even as a GP with a special interest in EDs and over 20 years experience of working with young adult patients in a university practice, I know this is not the case. There will be many patients who will go ‘under the radar’ and consequently not be offered any intervention. We know that EDs are ‘ hidden’ and it is worth reconsidering some of the barriers to disclosure on the patient's part as well as the barriers to detection on the part of the GP.

In addition to the sense of shame that frequently accompanies an ED, there is often a fear of the consequences of disclosure. Patients have articulated the fear that their coping strategy is going to be taken away and in the case of students or professionals in health care that their disclosure will result in suspension from their course or bar them from their chosen career. A lack of understanding of the rules of confidentiality deters others who believe that parents, schools, or academic departments could be informed. Fear of disapproval and a lack of confidence that the GP will know what to do (sometimes justifiably) may also impede the patient from seeking help.

From the GP's perspective, Glen has alluded to some of the barriers: time pressure and the fact that GPs are generalists, who for the most part have had little or no training in detecting or managing EDs. My impression is that most GPs will not go hunting for a disorder they lack confidence in managing and for which there is no clear care pathway. I have experience of colleagues expressing anxiety about managing eating-disordered patients and others denying that they have any such cases in their practice. Others seem to play ostrich and will concentrate on investigating the physical symptoms and signs referring patients to a series of inappropriate specialties without dealing with the underlying disorder. Hospital consultants are no better, obligingly investigating and discharging back to the GP without a mention of the patient's eating habits, vomiting, laxative abuse, or excessive exercise!

Given the barriers, how can we as GPs improve our detection rates? I like to think our role is to facilitate disclosure as much as it is to fine tune our antennae so that we pick up on the subtle hints our patients may be dropping. Patients are much more likely to disclose in an environment where there are clues that their ED will be understood. The EDs should be mentioned in practice leaflets, on websites, and sought in health questionnaires for new patient registration. Patients are invited to declare asthma, diabetes, depression so why not EDs? Posters in consulting rooms and patient information leaflets in waiting rooms and practice toilets could alert patients to services provided, reassuring patients that they will be able to discuss their problem in confidence. Empathy and warmth help most consultations but are particularly important when patients are ashamed and fearful of discussing their difficulties and asking for help. The GPs need to be able to work collaboratively with eating-disordered patients and discuss options for management. Ideally, the GP should have access to local guidelines on management, referral pathways, and an awareness of voluntary sector services. Clear guidance on the management of medical risk increases the GP's confidence in managing cases safely while waiting for specialist help or monitoring patients where care is shared between primary care and a specialist service. In practices where the prevalence is relatively high, it may be feasible to provide an ‘in house’ EDs clinic for mild to moderate cases providing Cognitive Behavioural Therapy or Guided Self-Help. In my experience once such a facility exists, the GPs’ case-findings improve considerably and if patients are allowed to self refer, those reluctant to see a GP will seek direct access.

Education for student doctors and GPs should clearly include detection and management of EDs. Nadia surmises that GPs are improving in their detection but we should not be dependent on the occasional GP with a special interest when every GP is likely to be encountering patients with EDs. Pattern recognition is integral to General Practice so an awareness of conditions likely to be ‘fronting’ EDs is also helpful; irritable bowel syndrome, multiple ‘food intolerance', and menstrual irregularities should all prompt questioning about eating. Opportunistic questioning in contraception clinics, new patient registrations where BMI is routinely documented and targeting patients with conditions such as depression, diabetes, and thyroid disorders would seem sensible.

With regard to screening, it is unlikely that there is a ‘one size fits all’ set of questions. Although I have diagnosed many patients with EDs, I have to admit I have never used a scripted set of questions. However, the questions suggested in the NICE guidelines would seem somewhat more useful than the SCOFF questionnaire for which we all remember the acronym but will have forgotten the actual questions.

In summary, GPs are not good at detecting and treating hidden disorders for which they perceive to be few treatment options. They can, however, help provide a setting where patients choose to disclose rather than hide their disorder. Given an appropriate education they can improve their detection and management and in collaboration with other health professionals, greatly improve the service given to these patients.
